# Suppression of Cellular Transformation by Poly (A) Binding Protein Interacting Protein 2 (Paip2)

**DOI:** 10.1371/journal.pone.0025116

**Published:** 2011-09-21

**Authors:** Amy B. Rosenfeld

**Affiliations:** Department of Microbiology & Immunology, Columbia University College of Physicians & Surgeons, New York, New York, United States of America; Victor Chang Cardiac Research Institute (VCCRI), Australia

## Abstract

Controlling translation is crucial for the homeostasis of a cell. Its deregulation can facilitate the development and progression of many diseases including cancer. Poly (A) binding protein interacting protein 2 (Paip2) inhibits efficient initiation of translation by impairing formation of the necessary closed loop of mRNA. The over production of Paip2 in the presence of a constitutively active form of hRas^V12^ can reduce colony formation in a semi-solid matrix and focus formation on a cell monolayer. The ability of Paip2 to bind to Pabp is required to suppress the transformed phenotype mediated by hRas^V12^. These observations indicate that Paip2 is able to function as a tumor suppressor.

## Introduction

Uncontrolled cell growth, the loss of contact inhibition and the ability of cells to grow in semi-solid matrix are all characteristics of transformed cells and the beginning of oncogenesis. These processes are dependent upon cell metabolism. Regulation of protein synthesis, a critical component of cell metabolism, is a critical component of cellular transformation and oncogenesis.

The cell regulates protein synthesis at many different steps, such as initiation of translation by a variety of mechanisms. One mean governs the recruitment of the ribosome, and the proteins required for initiation of translation to the RNA. Formation of the closed loop of mRNA is necessary for efficient translation initiation is also another point of regulation. The circularization of the mRNA brings together of the 5′- and 3′-ends of the mRNA. Several initiation proteins, and the poly (A) tail, a stretch of adenosine residues that varies in length found at the 3′-end of the most eukaryotic mRNAs, mediate formation of the closed loop. The eukaryotic initiation factor (eIF) complex 4F binds the 5′-end of the mRNA. Three proteins, eIF4E, the cap binding protein, eIF4G, a large scaffolding protein and the RNA bidirectional DEAD-box helicase eIF4A make up this complex. Within the amino-terminus (N-) of eIF4G is the binding site for eIF4E; thus, the complex is tethered to the 5′-end of the mRNA. The binding site for poly (A) binding protein (Pabp) lies within this region of eIF4G as well [1]. Papb also binds the poly (A) tail of the mRNA. Hence the interaction between eIF4E-eIF4G-Pabp links the two ends of the mRNA together, generating the closed loop [2], [3].

Two proteins, poly (A) binding protein interaction protein (Paip) 1 and 2, regulate the interaction between Pabp and the poly (A) tail, and Pabp and eIF4G [4], [5]. Although Paip1 and Paip2 are very similar including the Pabp interacting motifs (Pam) domains that bind Pabp between RNA recognition motifs (RRM) 2 and 3 and the Pabc domain found its carboxy terminus [6], [7], they have opposing functions. By interacting with Papb, eIF4A and eIF3, the initiation factor that binds the small ribosomal subunit bind and the central domain of eIF4G, aiding in the recruitment of the ribosome to the 5′-end of the mRNA, Paip1 is believed to stimulate translation in cultured cells [4], [8]. Paip2, in constrast, disrupts the closed loop of RNA necessary for efficient initiation. By directly binding Pabp, Paip2 facilitiates the dissociation of Pabp from the poly (A) tail, interrupts the Pabp-eIF4G interaction and prevents free Pabp in the cytoplasm from binding to poly (A) RNA [9]. Paip2 is therefore a negative regulator of protein synthesis.

The de-regulation and over production of many proteins involved in mRNA translation such as eIF4E has been implicated in cellular transformation and oncogenesis [10], [11], [12]. Although Papb is a highly abundant protein in the cytosol of the cell, its over production has also been correlated with the development of preleukemic thymuses in mice and gastrointestinal tumors in humans [13], [14]. Because it is a negative regulator of Pabp activity and of protein synthesis, Paip2 can therefore function as inhibitor of cellular transformation and a tumor suppressor.

## Results

**Figure 1:**
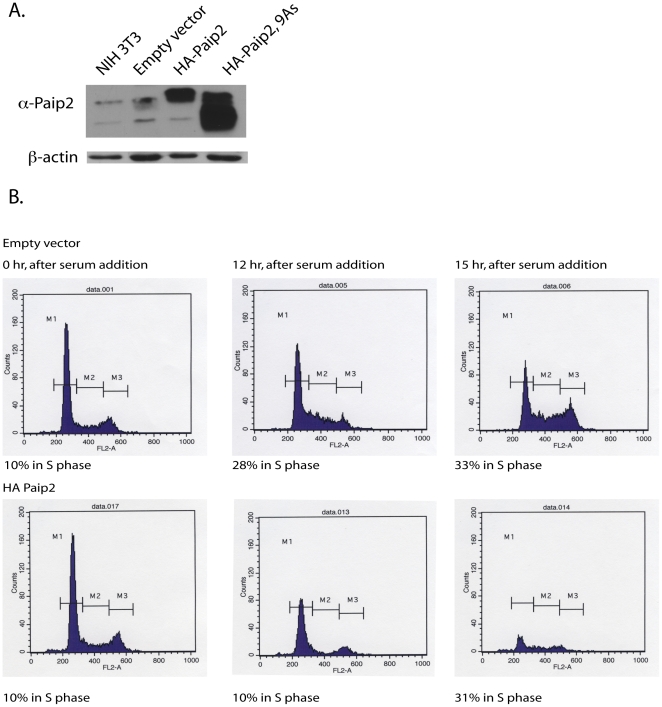
Cell cycle analysis of NIH 3T3 cells over producing Paip2. A. Western blot analysis of NIH3T3 cells alone, or infected with a retrovirus encoding for HA-Paip2 or HA-Paip2, 9As using anti-Paip2 antibody. B. Flow cytometry analysis demonstrating the 3-hour delay of entry into the cell cycle by BrUd staining of NIH3T3 producing wild type HA-Paip2 after the addition of serum following 72 hours of starvation.

Understanding the biological function of proteins can be investigated either by deleting the encoding gene or by the over production of the protein. To decipher how Paip2 alters the biology of the cell, the protein was over produced in NIH3T3 cells (Fig. 1A). Increased production of Paip2 did not significantly alter the growth properties of these cells as determined by flow cytometry. However, entry into the G1/S transition was delayed for several hours when cells synchronized by serum starvation were released from growth arrest (Fig. 1B). This observation suggests that Paip2 may regulate the translation of mRNAs whose protein products are necessary for the G1/S transition and correct control of the cell cycle.

**Figure 2:**
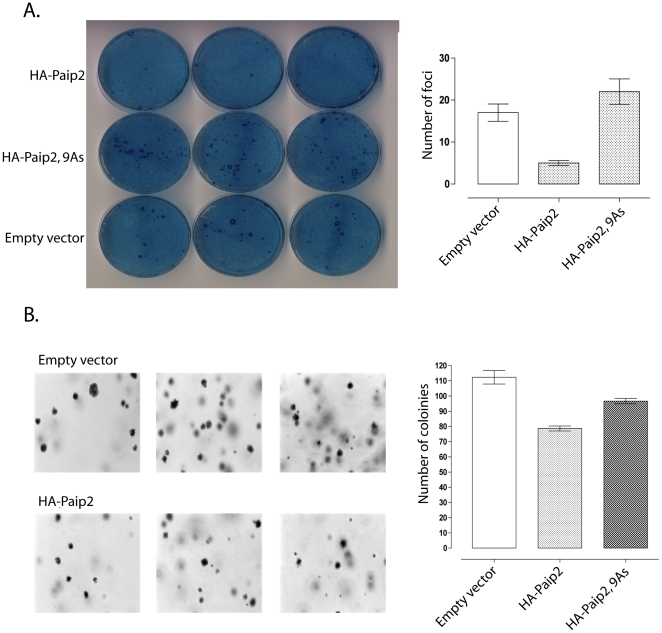
Paip2 impedes cellular transformation by hRas^V12^. A. Formation of cell focus by NIH3T3 cells producing hRas^V12^ and HA-Paip2 or HA-Paip2, 9As on a monolayer of NIH3T3cells. B. Impairment of anchorage independent cell growth of NIH3T3 cells producing hRas^V12^ by HA-Paip2.

Many proteins that govern progression through the cell cycle function either as oncogenes, by either promoting progression through the cycle or as tumor suppressors by restricting progression through the cell cycle. Therefore it is possible that Paip2 may function as a tumor suppressor as over production of the protein delays progression through the cell cycle. Paip2 was introduced into NIH3T3 cells producing a constitutively active form of ras, hRas^V12^ and assayed for the ability to form foci on a monolayer and growth in soft agar. Increased levels of Paip2 impeded the ability of hRas^V12^ producing cells from growing in soft agar and forming foci on a monolayer (Fig. 2A & B). These data suggest that Paip2 can function to inhibit cellular transformation.

**Figure 3:**
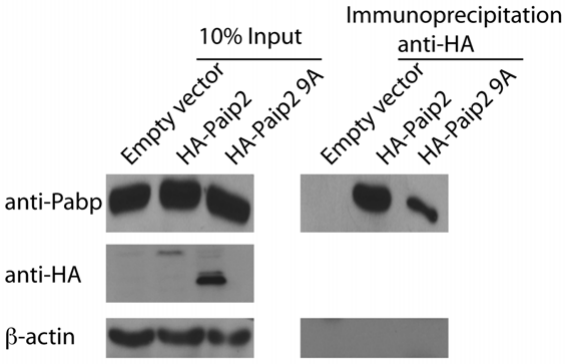
The Pam2 domain is required for the Paip2-Pabp interaction. Demonstration of the requirement for the Pam 2 domain of Paip2 for the Pabp-Paip2 interaction by immunoprecipation of HA-Paip2 or HA-Paip2, 9As from NIH3T3 cells by anti-HA antibody and Western blot analysis of cell lysates.

Aberrant regulation of protein synthesis has been implicated in the development and progression of cancer. Paip2 was initially identified as directly interacting with the initiation factor Pabp and impairing its ability to facilitate formation of the closed loop of mRNA necessary for efficient translation. The Pam domains, Pabp interaction motifs 1 and 2 within Paip2 have been described as mediating the Pabp-Paip2 interaction. However only the Pam2 domain is required for repression of translation by Paip2. It is believed that a series of glutamic acid residues within this region is required Pabp-Paip2 interaction (Fig. 3). Alteration of these glutamic acid residues to alanines not only impaired the Pabp-Paip2 interaction but it also partially ameliorated the ability of Paip2 to retard cellular transformation by hRas^V12^ (Fig. 2A &B). Therefore the ability of Paip2 to function as a tumor suppressor is dependent upon its ability to interact with Pabp.

## Discussion

Protein synthesis is critical to normal cell survival. Its de-regulation facilitates the development of many diseases including cancer. Therefore, any protein that modulates mRNA translation may function as either an oncogene or a tumor suppressor. Paip2 is a translation repressor. It inhibits translation initiation by promoting dissociation of Pabp from the poly (A) tail and eIF4G, and by preventing free Pabp from interacting with eIF4G, thereby disrupting formation of the closed loop of mRNA necessary to efficiently initiate translation. Alteration of the glutamic acids in the Pam2 domain of Paip2 reduced its ability to bind Pabp. The over production of wild type Paip2 suppresses cellular transformation mediated by the oncogene hRas^V12^. The over production of Paip2 in NIH 3T3 cells producing hRas^V12^ resulted in formation of fewer colonies in soft agar, and reduced formation of foci on monolayers. Moverover, the G1/M transition of cells that over produced Paip2 was delayed by several hours. These data are consistent with the observation that enhanced levels of Paip2 in *Drosophila melanogaster* also influences cell growth and proliferation. The over-production of dPaip2 impairs cell proliferation but not cell size in mitotically dividing cells of the wing disc. However, in non-dividiving cells of the eye and larval fat body, elevated levels of dPaip2 reduce cell size but not cell number [15]. These observations are similar to the phenotype of flies lacking the cell cycle regulator Cdk4 [16] and lead to the suggestion that the translation of the cdk4 encoding mRNA may be specifically sensitive to the intracellular concentration of dPaip2. Since, Paip2 is a phylogenetically conserved protein from flies to human, and functions as an inhibitor of translation initiation throughout evolution, it may be possible that translation of mRNAs responsive to the concentration of Paip2 would also be evolutionarily conserved. Identification of mRNAs whose translation is effected by the intracellular concentration of Paip2 should enhance our understanding of how mRNA translation regulates cell proliferation.

Formation of the closed loop of mRNA necessary for efficient translation initiation can be regulated at both the 5′-end and the 3′-end of the RNA. The translation inhibitors, eIF4E binding proteins (4E-BPs) prevent formation of the eIF4F complex at the 5′-end of the mRNA by preventing the eIF4G-eIF4E interaction [17], [18]. The serine-threonine kinase mammalian target of rapamycin (mTOR) regulates their activity [19]. The mRNAs whose translation is specifically sensitive to the intracellular concentration of the 4E-BPs and eIF4E have been well characterized, and include cyclin D1 [20], [21], [22], [23]. The 5′-end of these mRNAs is thought to contain stable secondary structures. Recruitment and scanning of the ribosome is therefore believed to be highly dependent upon eIF4E. It is possible that translation of this same population of mRNAs is regulated from the 3′-end by the Paip2-Pabp interaction. Translation of a different subset of RNAs, including those encoding Cdk4 and other proteins that regulate cell growth and proliferation, are more likely to be modulated by the Paip2-Pabp interaction. Neither Paip2 nor Pabp are regulated by mTOR and the aberrant activation of many different signaling cascades can result in cellular transformation. Identification of mRNAs whose translation is effected by the intracellular concentration of Paip2 should enhance our understanding of how mRNA translation regulates cell proliferation.

Previous work has demonstrated that the concentration of Paip2 within the cell is mediated by its interaction with Pabp. When not bound to Pabp, Paip2 can be targeted to the proteosome for degradation by ubiquitination by the E3 ligase EDD1. Activity of EDD1 is cell cycle regulated; it is high during G1/S and low during mitosis, suggesting that the level of Paip2 would be higher during mitosis than G1/S. Higher levels of Paip2 during mitosis may lead to in the dramatic reduction of 5′-end dependent initiation known to occur during this phase of the cell cycle. Aberrant degradation of Paip2 may lead to a higher concentration of free Pabp within the cytoplasm. Increased levels of Pabp correlate with the development of several cancers [13], [14]. Moreover, elevated levels of EDD1 have been observed in several tumors including breast, and ovarian [24]. The mechanism directly regulating degradation of Paip2 by EDD1 remains elusive. Activity of EDD1, however, is regulated by ERK2, a component of extracellular signal-regulated kinase pathway, in the presence of epidermal growth factor (EGF) [25]. Proteins such as B-RAF and Ras, known oncogenes, are known to regulate ERK2 activity. Thus it may be possible that ubiquitination of Paip2 by EDD1 may occur both in response to the presence of different growth factors and cytokines that stimulate ERK2 activity and also in cells in which B-Raf and Ras are constitutively active. Continued understanding how of the interactions of Paip2 and Pabp, and of Paip2 and EDD1 are regulated should augment our understanding of how efficient translation initiation is controlled and promote the development of novel anti-cancer therapeutic agents.

## Materials and Methods

### Cells and plasmids

NIH3T3 cells were grown in Dulbecco's modified Eagle medium (Sigma) 10% bovine calf serum (Invitrogen) and 1% penicillin-streptomycin (Invitrogen). Selection of NIH3T3 cells stably expressing hRas^V12^ was maintained by the addition of puromycin at every passage. NIH3T3 cells stably expressing murine HA-Paip2 or HA-Paip2 (9A) were grown in the presence of hygromycin. NIH3T3 cells stably producing HRas^V12^ and HA-Paip2 or HA-Paip2 (9A) were maintained by the addition of puromycin and hygromycin at every passage. Phoenix-293-T cells were grown in Dulbecco's modified Eagle medium, 10% fetal calf serum and (Invitrogen) and 1% penicillin-streptomycin (Invitrogen). Plasmids p-BABE hRas^V12^ and pWzl were generous gifts of Scott Lowe, Cold Spring Harbor Laboratory, NY.

### Generation of stable cell lines

Cell lines over producing of hRas^V12^, HA-Paip2 and HA-Paip2 (9A) were generated by introducing the appropriate encoding plasmid into phoenix-293-T cells by calcium phosphate transfection. Viruses were harvested 48 hours post-transfection and filtered (0.45 mm-pore-size filter). NIH3T3 cells were infected with the appropriate virus or viruses and 48 hours postinfection were selected by the addition of puromycin, hygromycin or puromycin and hygromycin. Production of hRas^V12^, HA-Paip2 and HA-Paip2 (9A) was confirmed by Western blot analysis.

### Co-immunoprecipation and western blot analysis

Cells were collected following stimulation and lysed in cold buffer containing 20 mM Tris-HCl (pH 7.5), 0.1 mM EDTA, 1 mM DTT, 100 mM KCl, and 10% glycerol (v/v), and a mixture of protease inhibitors (Roche Diagnostics). Protein content was determined using the Bio-Rad Protein Assay (Mississauga, ON, Canada). Cellular extracts (1 mg of protein) were incubated end-over-end with 50% slurry of protein G-sepharose for 1 hour at 4°C, and with mouse monoclonal anti-HAII antibody overnight at 4°C. The mixture was centrifuged at 3000 rpm for 30 seconds and the resin was washed three times in 0.5 ml of the above buffer. Proteins were eluted with 2× Laemmli sample buffer and resolved by 15% SDS-PAGE. The separated proteins transferred onto a nitrocellulose membrane (Protran, Perkin-Elmer). Membranes were incubated at room temperature in 5% nonfat milk dissolved in blot buffer (10 mM Tris, pH 7.5, 150 mM NaCl, 0.05% Tween 20) for 1 hour and incubated at 4°C overnight with one of the following specific antibodies: anti-HAII, anti-Pabp and anti-β-actin was from Sigma-Aldrich. Proteins were then detected with anti-mouse (sheep) or anti-rabbit (donkey) IgG horseradish peroxidase-linked antibodies (GE Healthcare, Baie d'Urfe, QC, Canada) and subsequent visualization by Western Lightning-chemiluminescent substrate system (Perkin Elmer).

### Cell cycle analysis

NIH3T3 cells and NIH3T3 cells producing HA-Paip2 and HA-Paip2 (9A) were grown to 40% confluency in presence 10% bovine calf serum. Cells were washed twice with 1× PBS and grown for 72 hours in presence of 0.1% bovine calf serum in order to synchronchrous their position within the cell cycle. Cells were harvested at various time after the addition of medium containing 10% bovine calf serum, washed twice in cold 1× PBS, fixed in ethanol, stained with prodium iodine, and analyzed by flow cytometry.

### Cell transformation assays

#### Soft agar assays

Approximately 5×10^4^ cells were resuspened in 2.5 ml of 0.35% (wt/vol) agar solution, containing DMEM plus 20% BCS and onto a 0.5% (wt/vol) agar solution containing DMEM plus 20% BCS in a 60-mm plate. 2 days after incubation, 3 ml of DMEM supplemented with 20%BCS was added. Colonies were counted 21 days after plating.

#### Focus formation on a cell monolayer

NIH3T3 cells stably producing HRas^V12^ and HA-Paip2 or HA-Paip2 (9A) were on a monolayer of NIH3T3 in 1000∶1 ratio for 28 days. Foci were fixed in methanol and stained by methylene blue.

All data are the mean ± the standard of error of the mean from three independent experiments.
